# Incorporation of Mometasone Furoate into a Cyclodextrin Metal-Organic Framework to Optimize Nasal Administration

**DOI:** 10.3390/pharmaceutics17060788

**Published:** 2025-06-17

**Authors:** Jie Li, Yuhua Guo, Yan Liu, Qingfang Gao, Siwen Wang, Li Wu, Caifen Wang, Xiaohong Ren, Jiwen Zhang

**Affiliations:** 1 Biological and Pharmaceutical Sciences, Wuya College of Innovation, Shenyang Pharmaceutical University, Shenyang 110016, China; 13194759625@163.com; 2 Yangtze Delta Drug Advanced Research Institute, Nantong 226126, China; wuli@simm.ac.cn; 3 Shanghai Institute of Materia Medica, Chinese Academy of Sciences, Shanghai 201210, China; 15935107669@163.com (Y.G.); wangsiwen@simm.ac.cn (S.W.); fencaiwang@163.com (C.W.); renxh2015@163.com (X.R.); 4 School of Pharmacy, Shenyang Pharmaceutical University, Shenyang 110016, China; yanll197@163.com (Y.L.); gqf4g728@163.com (Q.G.); 5 State Key Laboratory of Quality Research in Chinese Medicine, Macau University of Science and Technology, Macau 999078, China

**Keywords:** mometasone furoate, cyclodextrin metal-organic framework, nasal powder spray, three-dimensional biomimetic model of the human nasal cavity

## Abstract

**Background/Objectives:** Mometasone furoate (MF) is a topical corticosteroid used to reduce allergic and inflammation symptoms. In this study, MF was incorporated into the hydrophobic cavities of γ-cyclodextrin metal-organic frameworks (CD-MOFs) to prepare MF@MOF powders for nasal delivery. **Methods**: MF@MOF particles were characterized by scanning electron microscopy (SEM), powder X-ray diffraction (PXRD), Fourier transform infrared spectroscopy (FTIR), and thermogravimetry. A transparent biomimetic model of the human nasal cavity was produced by 3D printing and used to evaluate intra-nasal depositions patterns. **Results**: Drug loading was optimized by incubating MF with a CD-MOF at a ratio of 4% for 1 h at 40 °C, and the cubic morphology and particle size of the nanoparticles were not altered using an incubation method. PXRD and FTIR analyses confirmed the successful loading of MF into the CD-MOF. Using a 3D biomimetic nasal cavity model, a 30° administration angle was found to result in reduced drug accumulation in the nasal vestibule and enhanced deposition in the respiratory and olfactory regions, compared with administration at 45°. Approximately 51% of the drug reached the respiratory zone in the model of the nasal cavity from male subjects, while almost 60% of the drug reached this zone in the model associated with female subjects. Compared with nasal sprays, nasal powder sprays had less deposition in the nasal vestibule and more deposits in the middle and inferior nasal concha. **Conclusions**: MF@MOF is suitable for intranasal administration. Delivery of MF as a nasal powder shows potential in the treatment of chronic rhinosinusitis.

## 1. Introduction

Chronic rhinosinusitis (CRS) is a persistent inflammatory disorder (>12 weeks) affecting nasal and paranasal sinus mucosa, characterized by nasal obstruction, rhinorrhea, postnasal drip, and potential eye diseases [1,2,3], with a global prevalence ranging from 4.8% in Chinese cities to 11% in Europe [4,5,6,7]. Clinically, CRS can be divided into two types, with and without nasal polyps [8]. CRS can be treated with drug therapy or surgery [9].

**Figure 1:**
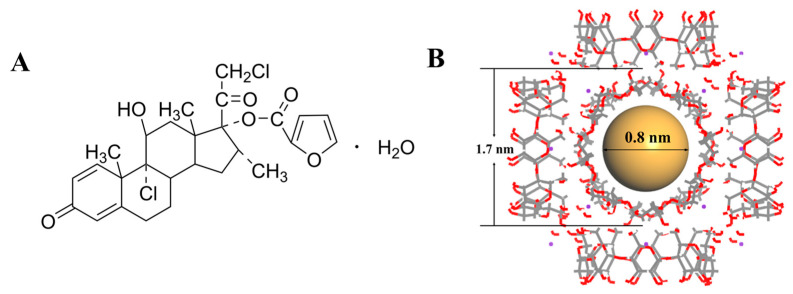
The chemical structure of mometasone furoate (**A**). Schematic representation of γ-CD-MOF crystalline structure. The gray solid lines represent C–C bonds, red solid lines denote C–O bonds, purple dots indicate K⁺ ions, and brown spheres illustrate the volumetric dimensions of CD-MOF’s hydrophobic cavities (**B**).

In recent years, corticosteroids have been employed as first-line therapeutic agents for CRS, particularly for nasal polyp management. Mometasone furoate (MF), a synthetic glucocorticoid as shown in Figure 1A, demonstrates potent anti-inflammatory and anti-allergic effects [10,11,12]. However, MF is classified as a Biopharmaceutics Classification System (BCS) Class II drug [13], and its clinical utility is constrained by poor solubility characteristics. To overcome this limitation, we attempted to develop a novel delivery system using γ-cyclodextrin metal-organic frameworks (CD-MOFs). These crystal cubes have a high specific surface area and nanoscale molecular structure, as shown in Figure 1B. Drug molecules confined within the nanochannels of the MOF may exist in an amorphous or metastable state, which typically exhibits higher solubility than their crystalline counterparts [14,15]. The highly ordered mesh porous structure of CD-MOFs has found applications in multiple fields, including materials science [16], drug delivery [17], and environmental remediation [18].

Nasal administration offers distinct advantages, including rapid local action and avoidance of hepatic first-pass metabolism, making it ideal for anti-inflammatory therapies [19,20,21]. In contemporary practice, the nasal route of administration is commonly utilized for managing localized upper respiratory pathologies, encompassing nasal congestion, infectious rhinitis, and allergic nasal conditions. With ongoing developments, nasal administration has become the preferred route for vaccines [22,23], systemic drugs [24,25], and “nose to brain” [26,27,28]. Nevertheless, physiological barriers such as mucociliary clearance and excipient toxicity can significantly impede the intranasal delivery of therapeutic agents [29,30]. To address these dual challenges, we employed 3D-printed biomimetic nasal models for delivery optimization. Three-dimensional printing technology (3DP) was used to produce a transparent model of the human nasal cavity, a process that allowed for the additive manufacturing of complex structures through sequential deposition, thereby accommodating virtually any shape and size [31,32,33,34,35,36,37]. Therefore, we developed biomimetic models of the human nasal cavity using 3D printing technology to investigate nasal drug delivery.

This study involved the encapsulation of MF within the hydrophobic cavity of CD-MOFs to fabricate MF@MOF microparticles. The synthesis protocol of CD-MOFs is described, followed by comprehensive characterizations of the carrier particles and the resulting inclusion complex, and biomimetic models of the human nasal cavity were used to characterize drug deposition upon nasal administration of MF@MOF. This dual approach synergized nanocarrier engineering with advanced biomimetic modeling to optimize CRS treatment strategies.

## 2. Materials and Methods

### 2.1. Materials

Mometasone furoate was purchased from Shandong Sirui Pharmaceutical Co., Ltd. (Heze, China). Mometasone furoate nasal spray was purchased from Zhejiang Xianju Pharmaceutical Co., Ltd. (Taizhou, China). γ-Cyclodextrin (γ-CD) was purchased from MaxDragon Biochem Ltd. (Guangzhou, China). Methanol, potassium hydroxide (KOH), poly (ethylene glycol)-20000 (PEG20000), ethanol, glacial acetic acid, and other reagents of analytical grade were purchased from Sinopharm Chemical Reagent Co., Ltd. (Shanghai, China), and used without further purification.

### 2.2. Synthesis of CD-MOFs

KOH (8.65 g) and γ-CD (25.00 g) were weighed at a molar ratio of 1:8 and dissolved in deionized water (772 mL) at room temperature. After the solids were dissolved, methanol (460 mL) was added, and the mixture was incubated at 70 °C until the solution became clear within 30 min. PEG20000 (2.46 g) was added, followed by stirring for 20 min. The solution was subsequently cooled in an ice-water bath and incubated overnight.

For neutralization, the CD-MOFs were suspended in 150 mL ethanol, and glacial acetic acid (2.8 mL) was added. The mixture was stirred to ensure complete neutralization. The neutralized precipitates were collected by vacuum filtration and dried at 70 °C for 3 h.

### 2.3. Characterization of CD-MOFs

The morphologies of basic and neutralized CD-MOFs were observed under a polarizing microscope (DYP-990C, Dianying, Shanghai, China). The surface morphology of CD-MOFs was characterized through SEM (FlexSEM1000, Hitachi, Japan). The samples were covered with a thin layer of gold and observed at an appropriate magnification. The particle size distribution was determined using a dry laser diffraction particle size analyzer (HELOS, Sympatec GmbH, Clausthal-Zellerfeld, Germany). The powder samples were uniformly dispersed in air using a RODOS dispersion system under a dispersion pressure of 3 bar and a flow rate of 50 L/min. The crystal structures of unloaded basic or neutralized CD-MOFs were characterized by powder X-ray diffraction (PXRD) using a Bruker D8 Advance diffractometer (Bruker, Billerica, MA, USA).

### 2.4. Drug Loading of MF into CD-MOFs

**Figure 2:**
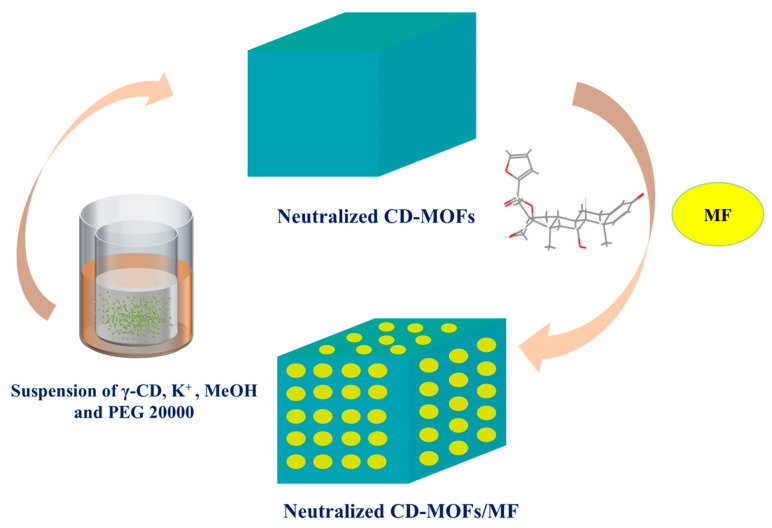
Schematic illustration of the synthesis of CD-MOF-Micro and MF-loaded CD-MOFs.

MF was loaded into neutralized CD-MOFs by incubating CD-MOFs in an MF-containing solution. The detailed schematic of the synthesis process is illustrated in Figure 2. Briefly, a sample (210 mg) of dried, neutralized, micron-sized CD-MOF ([K_2_(C_48_H_80_O_40_)(OH)_2_]n, MW = 1409.34) [38] powder was dispersed ultrasonically into 5 mL of a 2 mg/mL solution of MF in an organic solvent (acetone, ethyl acetate, or ethanol) to achieve an 8:1 molar ratio of CD-MOFs to MF. The mixture was incubated at 40 °C with stirring (200 rpm) for 1 h. The mixture was dried in a rotary evaporator to obtain MF-loaded neutralized CD-MOFs. For each solvent, three different molar feed ratios of CD-MOFs to MF (8:1, 6:1, and 4:1) were investigated in a gradient manner.

To determine the MF loading amount in MF@MOF, 10 mg MF@MOF sample was weighed and dissolved in a methanol–water mixture (75:25, *v*/*v*) within a 5 mL flask. The resulting solution was centrifuged (12,000× *g* rpm, 5 min; Micro 2R, Thermo Fisher Scientific, Waltham, MA, USA), and the supernatant was analyzed by high-performance liquid chromatography (HPLC). The drug loading capacity was calculated using Equation (1).

(1) $$\mathrm{Payload}\;(\%)=\;\frac{\mathrm{Mass}\;\mathrm{of}\;\mathrm{MF}\;\mathrm{loaded}\;\mathrm{in}\;\mathrm{CD}\text{-}\mathrm{MOF}}{\mathrm{Mass}\;\mathrm{of}\;\mathrm{MF}@\mathrm{MOF}}\times 100$$

The quantification of MF was performed by HPLC (Agilent 1290, Agilent Technologies, Palo Alto, CA, USA) equipped with a diode-array detector (DAD, GA4212A, Gilson, Villiers-le-Bel, France). Separation was achieved using an InerSustain AQ-C18 column (250 mm × 4.6 mm, 5 μm) (GL Sciences, Shanghai, China) maintained at 40 °C. The mobile phase, composed of methanol (A) and 0.2% aqueous acetic acid (B) (75:25, *v*/*v*), was delivered at a flow rate of 1.0 mL/min. Detection was carried out at 254 nm with an injection volume of 20 μL. Data acquisition and analysis were conducted using ChemStation software (C.01.07, Agilent).

### 2.5. Morphology and Other Characterizations of MF@MOF

The morphological structures of MF@MOF variants were investigated by field emission scanning electron microscopy (SEM, SU7000, Hitachi, Japan). Fourier transform infrared spectroscopy (FTIR) is an instrumental technique employed for the analysis of functional groups in chemical compounds. In this study, a spectrometer (Nicolet FTIR 6700, Thermo Fisher Scientific, Waltham, MA, USA) was utilized to examine the functional group structures of chemical substances. Prior to analysis, the samples were thoroughly dried with potassium bromide (KBr). Subsequently, 1–2 mg of each sample was homogenously mixed with KBr at a ratio of 1:100. The mixture was meticulously ground and then compressed into pellets. Infrared absorption spectra of the samples were acquired within the wavenumber range of 4000 to 400 cm^−1^. Thermogravimetric analysis (TGA) was conducted using a thermal analysis system (Pyris TGA, PerkinElmer, Waltham, MA, USA) at a heating rate of 10.00 °C/min under a constant nitrogen flow of 20 mL/min. The sample (approximately 5 mg) was loaded into an aluminum pan, and the weight loss (%) of the sample was measured upon heating from 30 to 600 °C.

### 2.6. Solubility Studies In Vitro

The apparent solubility of MF and MF@MOF in pH 1.2, pH 6.8, distilled water, and artificial nasal solution was determined. Artificial nasal solution was composed of 8.761 g NaCl, 2.982 g KCl, 0.555 g CaCl_2_, and 1.211 g Tris. The volume of the solution was fixed to 1 L, and the pH was adjusted to 6.3 after being dissolved in deionized water [36]. Excess MF (10 mg for MF raw material) and above drug-loading samples (about 50 mg drug content for MF@MOF) were placed in plastic centrifuge tubes, and 1 mL water was added to prepare the supersaturated solution, which was placed in a constant temperature incubation shaker (ES-60p, Wiggens, Wuppertal, Germany) for 200 rpm at 25 °C for 2 h. The suspension was filtered through a 0.45 μm membrane to eliminate any insoluble particles. The solubility of MF in different media was evaluated by HPLC. The analysis was conducted according to the conditions in Section 2.4. The solubility of each sample was measured in triplicates.

### 2.7. In Vitro Release Study of MF@MOF

The solubility of MF and its release from the particles were determined according to a method described in the Chinese Pharmacopoeia, Volume IV (2020). The following aqueous buffers were prepared: 0.1 M HCl (pH 1.2), 100 mM phosphate (pH 6.8), and an artificial nasal solution. Free MF, MF@MOF, and MF directly mixed with CD-MOFs were placed in 5 mL media. These mixtures were incubated at 37 °C with stirring (50 rpm). Samples (1 mL) were withdrawn at 5, 10, 20, and 30 min after the initiation of the experiment, and the liquid was replaced with an equal volume of fresh medium. Insoluble particles were removed by filtration through a 0.22 μm membrane, and the presence of MF was determined by quantitative HPLC. Curves of drug release as a function of time were prepared, and the cumulative release was calculated. Analysis was conducted according to the conditions in Section 2.4. All experiments were repeated three times. Owing to the immediate replenishment of fresh medium following each sampling, the cumulative release percentage was determined by the direct summation of the drug concentrations measured at all sampling intervals. The cumulative release percentage was calculated using Equation (2).

(2) $$R_{\;\mathrm{cum}}\;(\%)=\frac{\sum_{i=1}^{n}\mathrm{Ci}\;\times \;\mathrm{Vsample}}{W}\;\times \;100$$

C_i_: the drug concentration at the i-th sampling;

V_sample_: sampling volume;

W: mass of MF loading (in MF@MOF).

### 2.8. Application of 3D Biomimetic Human Nasal Cavity Models

**Figure 3:**
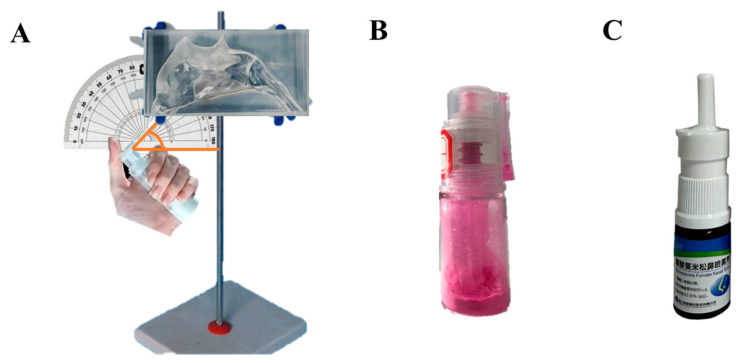
Photograph of a 3D biomimetic model of the female nasal cavity. The drug (MF powder or commercially available MF spray) was administered through the nostril with an angle of either 30° or 45° (**A**). Physical diagram of the device of the nasal powder spray dispenser (**B**). Photograph of the commercially available mometasone furoate nasal spray device (**C**).

The biomimetic human nasal cavity model was developed based on head and neck computed tomography (CT) scans from 32 Chinese adults (16 males and 16 females, aged 20–59 years), utilizing three-dimensional reconstruction and additive manufacturing technologies with transparent photopolymer resin as the primary construction material. The modeling work was principally conducted by our research team [39]. In order to prevent the entrainment phenomenon caused by particle rebound during the test, the interior surface of the model was pre-treated with a dimethicone coating. The procedure was performed by slowly wetting the inner surface of the model with a dimethicone-saturated cotton swab. The model surface was pre-wetted with dimethicone to facilitate visual monitoring of powder deposition as the sprayed powder would otherwise not adhere directly to the surface. Given the inherent sex differences in humans, separate models for males and females were employed to ensure data representativeness. The model was stabilized using a tripod mount, with a goniometer utilized to verify and maintain precise spray angle alignment of the administration nozzle (Figure 3A). The upper and lower parts of the model were fastened securely using petroleum jelly. The nozzles of the nasal powder dispenser were extended to a depth of 10 mm into the nasal cavity at conical angles of 45° or 30°, and the powder was extruded ten times. The physical diagram of the device of mometasone furoate nasal powder spray is shown in Figure 3B. The nasal cavity model was placed under a 254 nm UV analyzer (WFH-203B, Hangzhou Qiwei Technology Co., Ltd., Hangzhou, China) because MF@MOF exhibited orange fluorescence at a wavelength of 254 nm. The photos were taken at a fixed camera location by placing the nasal cavity model on an inclined plane with a slope of 10 to obtain a parallel image to the lens. The photosensitivity was set to 1600, and the shutter speed was set to 2 s in the original model to ensure the consistency of the photo parameters. ImageJ (v1.8.0) was used to assess the average fluorescence intensity in a semi-quantitative manner, and the drug distribution throughout the nasal cavity model was obtained. The deposition behavior of the MF@MOF was compared with that of a commercially available MF nasal spray (Figure 3C). The commercially obtained mometasone furoate nasal spray delivered a dose of 50 μg per spray.

## 3. Results

### 3.1. Morphology and Characterization of CD-MOFs

**Figure 4:**
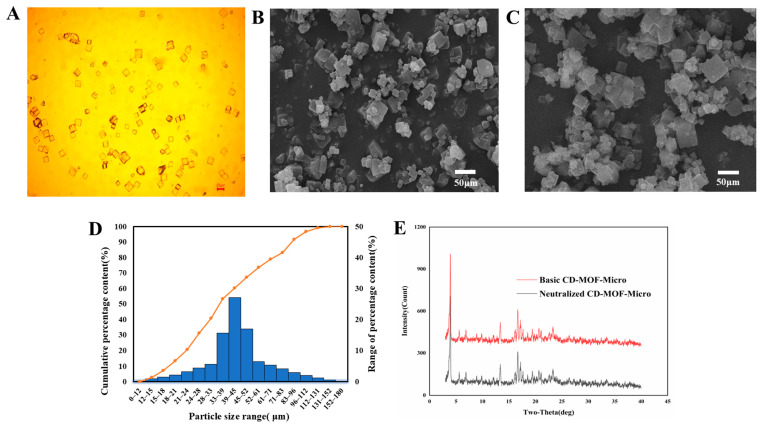
Characterization of CD-MOF particles. The particles were observed under polarizing microscopy (**A**). The sizes of basic CD-MOF-Micro particles were analyzed using SEM (**B**). The morphology of neutralized CD-MOF-Micro were analyzed using SEM (**C**). Distribution of grain sizes of basic CD-MOFs (**D**). CD-MOF powders were analyzed by PXRD before (Basic CD-MOF-Micro) and after (Neutralized CD-MOF-Micro) neutralization with acetic acid (**E**).

Polarizing microscopy imaging of the prepared CD-MOF particles demonstrated that they were of a uniform size (Figure 4A). The specific sizes of the basic CD-MOF-Micro and neutralized CD-MOF-Micro were determined by SEM (Figure 4B,C). As shown in a histogram of the particle size distribution (Figure 4D), the particle sizes ranged from 12 to 152 μm, with most particles falling in a size range from 24 to 83 μm. Specifically, the D10, D50, and D90 values were 16 μm, 42 μm, and 96 μm, respectively. The CD-MOF particles were produced in a basic solution, which was then neutralized with acetic acid. To determine whether the acetic acid treatment altered the particle structure, we analyzed the basic and neutralized CD-MOFs using PXRD (Figure 4E). We found that the pattern of peaks obtained upon analysis of the basic CD-MOF particles was not different from that of neutralized CD-MOFs, indicating that acidification with acetic acid did not affect the crystal pattern of CD-MOFs.

### 3.2. Optimization of Drug Loading Conditions

First, we investigated the effects of solvent on the loading of MF into the CD-MOF particles. As shown in Table 1, the loading efficiency was similar when the drug was applied in acetone, ethyl acetate, or ethanol. As it was the most environmentally safe among the three solvents, anhydrous ethanol was selected as the optimal drug carrier solvent. The drug loading was controlled at 4% through the incubation method, and the feeding ratio was optimized. At present, the drug loading capacity of the prepared MF@MOF was controlled at about 4%. The optimal conditions for loading mometasone furoate in the incubation method were as follows: the loading solvent was anhydrous ethanol, the MF feeding ratio was 4%, and heating was performed at 40 °C for 1 h.

### 3.3. Characterization of MF@MOF

**Figure 5:**
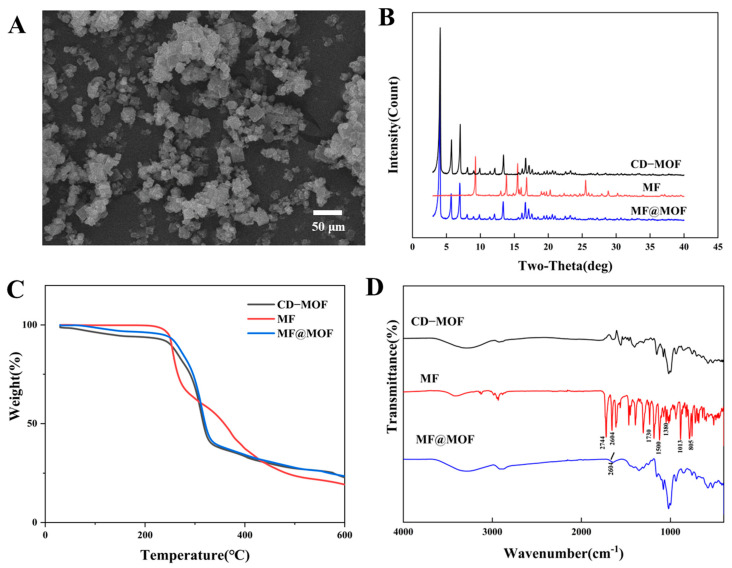
Scanning electron microscopy analyses of CD-MOFs before and after loading. (**A**) MF@MOF. Structural analyses of MF and the nanoparticulate preparations. (**B**) PXRD patterns. (**C**) TGA thermographs. (**D**) SR-FTIR spectra.

According to the SEM analysis, there were no significant morphological changes in CD-MOFs before and after drug loading (Figure 4C and Figure 5A). The comparative study suggested that the morphology of CD-MOFs was not affected detectably by the drug loading process.

In the PXRD spectrum (Figure 5B), MF exhibited sharp diffraction peaks at 9.3°, 13.8°, 15.5°, 16.8°, and 25.5°, indicating that the drug was crystalline. The diffraction peaks of CD-MOFs at 4.04°, 5.70°, 6.97°, 13.36°, 16.63°, and 17.13° similarly indicated successful crystallization of CD-MOFs. The spectrum of MF@MOF exhibited similar peaks to that of CD-MOFs. However, the MF@MOF spectrum lacked the characteristic peaks of MF at 9.3° and 25.5°. The disappearance of characteristic crystalline peaks of the drug could generally be attributed to three possible reasons: (1) The drug loading was too low to be detected by the PXRD; (2) the crystalline form had transformed into an amorphous state, or strong interactions existed between the drug and CD-MOFs; or (3) the drug had been successfully encapsulated within the MOF cavities. Therefore, additional characterization was required to further verify the exact mechanism.

The decomposition of MF, CD-MOFs, and MF@MOF as a function of temperature was investigated using TGA. As shown in Figure 5C, the mass of the MF sample rapidly decreased within the temperature range of 247 to 520 °C. However, the analysis showed that the degradation of CD-MOFs occurred between 45 and 221 °C, while that of MF@MOF occurred between 260 and 308 °C. As these changes corresponded to the volatilization of water and the cracking of CD-MOFs, the differences in temperature ranges provided evidence for physical interactions between MF and CD-MOFs. The trends of the curves for CD-MOFs and MF@MOF were essentially identical. This phenomenon may be attributed to the insufficient drug loading quantity, which necessitates further verification through complementary characterization techniques for a comprehensive assessment.

A comparison of SR-FTIR spectra of CD-MOFs, MF, and MF@MOF is shown in Figure 5D. In the spectrum of MF, the presence of absorption peaks at 2744 cm^−1^, 2604 cm^−1^, 1500 cm^−1^, and 1380 cm^−1^ confirmed that a methyl group was present in the structure, while the peak at 805 cm^−1^ was assigned as the stretching vibration peak of the chlorine atom. The peak at 1730 cm^−1^ was the stretching vibration peak of C=O, and the peak at 1013 cm^−1^ was the stretching vibration peak of C–O–C. Comparative analysis of the Fourier transform infrared (FTIR) spectra between MF@MOF and CD-MOFs revealed the emergence of new characteristic bands, which could be attributed to C–H stretching vibrations (2604 cm^−1^). The presence of these distinctive spectral features, when compared with the unique characteristic bands of pure MF, provided evidence for the successful incorporation of MF within the MF@MOF composite.

### 3.4. Enhanced Solubility of MF@MOF

**Figure 6:**
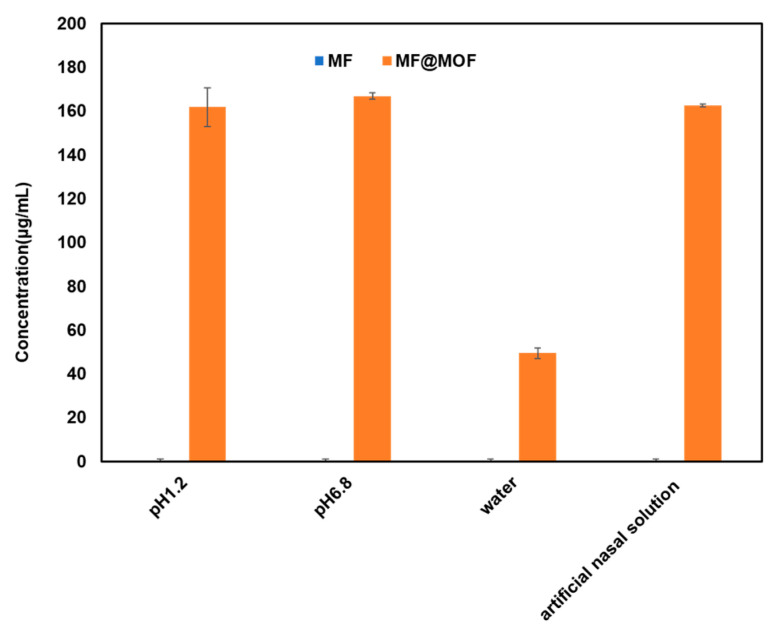
The solubility of MF and MF@MOF in four media.

The MF compound was almost insoluble in water. The quantification limit of MF determined by HPLC was 0.2 μg/mL. Its solubility in HCl solution (pH 1.2), phosphate buffer (pH 6.8), water, and artificial nasal solution (pH 6.5) was below the detection limit (0.2 μg/mL) as the current HPLC method failed to detect it. As shown in Figure 6, the solubility of MF@MOF significantly increased in these media. The minimum solubility of MF in water after CD-MOF encapsulation reached 49.5 μg/mL, while values of 161.8 μg/mL in HCl solution (pH 1.2), 166.8 μg/mL in phosphate buffer solution (pH 6.8), and 162.5 μg/mL in artificial nasal solution were observed. These results demonstrated that the solubilization effect of CD-MOFs on MF was pH independent.

### 3.5. In Vitro Evaluation of Drug Release

**Figure 7:**
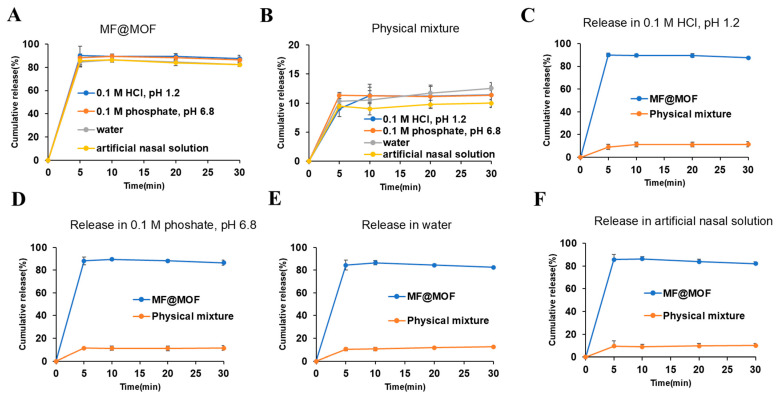
In vitro release profiles of MF@MOF and physical mixture in HCl solution (pH 1.2), phosphate buffer solution (pH 6.8), water, and artificial nasal solution. (**A**) MF@MOF release profile in media at 37 °C for up to 30 min. (**B**) Physical mixture of MF and CD-MOF release profile in media at 37 °C for up to 30 min. (**C**) MF@MOF and physical mixture release profiles in HCl solution (pH 1.2). (**D**) MF@MOF and physical mixture release profiles in phosphate buffer solution (pH 6.8). (**E**) MF@MOF and physical mixture release profiles in water. (**F**) MF@MOF and physical mixture release profiles in artificial nasal solution. MF was quantified by HPLC assay. Concentration of MF expressed as a percentage of the initial MF loaded in the CD-MOF (means ± SD, *n* = 3).

Drug release was characterized by incubating drug-loaded particles in buffers of different pH and then using quantitative HPLC to measure the release of drug into the buffer over time. It is worth noting that the cumulative release amount of MF@MOF exhibited a significant volume-dependent behavior: approximately 90% was released in 5 mL medium (Figure 7A). Additionally, the physical mixture of MF and the CD-MOF released only about 10% under all conditions (Figure 7B), which was attributed to the rapid precipitation and dissolution of the unencapsulated MF due to its low solubility (less than 0.2 μg/mL in all media). As shown in Figure 7C–F, the release profiles of both MF@MOF and the physical mixture reached a plateau phase after 5 min. More precisely, the cumulative drug release profiles revealed significant differences between formulations across all test media. In the HCl solution (pH 1.2), MF@MOF achieved 87.5% ± 2.79% release within 30 min, compared with merely 11.4% ± 0.87% for the physical mixture. Similar trends were observed in phosphate buffer solution (pH 6.8) (86.4% ± 2.29% vs. 11.4% ± 2.15%), in water (82.4% ± 0.86% vs. 12.6% ± 0.38%), and in artificial nasal solution (82.2% ± 1.71% vs. 10.0% ± 0.66%), consistently demonstrating the superior release performance of the MF@MOF formulation (Figure 7C–F).

The significant difference between MF@MOF and the physical mixture revealed the crucial role of MOF encapsulation in enhancing drug dispersion and maintaining release kinetics. The above results collectively indicated that the CD-MOF overcame the solubility limitation of MF—this property holds significant application value in scenarios requiring local delivery (such as low-volume nasal administration vs. a high-volume gastrointestinal environment).

### 3.6. Deposition of MF Powder in a 3D Biomimetic Human Nasal Cavity Model

#### 3.6.1. Delivery of MF@MOF with a Nozzle Angle of 45°

**Figure 8:**
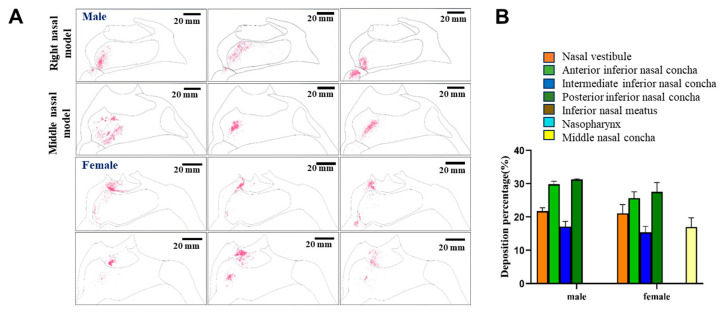
Nasal cavity deposition pattern of MF delivered as a nasal powder with an injection angle of 45°. (**A**) The drug was delivered into 3D models mimicking the nasal cavities of male or female human subjects, and deposition of fluorescent MF was observed under UV irradiation. (**A**) Semi-quantitative analysis of the fluorescence intensities shown in (**B**).

Delivery of the drug within the nasal cavity under various conditions was determined using transparent models that mimicked the typical nasal cavities of male or female human subjects. Drug preparations were delivered using nozzles oriented at specific angles, and the distribution of the MF, which was naturally fluorescent, was imaged under UV irradiation. As shown in Figure 8A, when delivered under the external force of the device with the nozzle oriented at a 45° angle, the drug powder was mainly delivered to the anterior area of the nasal cavity, including the nasal vestibule, whether the nozzle was placed within the turbinate region or near the nasal septum.

The distribution of the drug was analyzed semi-quantitatively by determining the average fluorescence intensity within each region (Figure 8B). Interestingly, in the model that represented the nasal cavities of females, with the nozzle oriented at a 45° angle, 16.97% of the powder reached the middle turbinate region, while no detectable powder reached the middle turbinate of the model that represented the nasal cavities of males. However, the proportions of the drug to reach the anterior and posterior regions of the nasal cavity were predicted to be approximately 5% higher in males than in females. The proportion of the drug reaching the middle part of the lower turbinate region was not significantly different between the two models. Overall, with the nozzle at a 45° angle, in the male model, approximately 48.43% of the drug reached the respiratory area, including the middle and posterior inferior turbinate regions, while approximately 42.94% of the drug reached the respiratory area in the female model. The drug exhibited no detectable distribution in either the inferior nasal meatus or the nasopharyngeal region. As noted above, significantly more drug reached the deeper areas of the nasal cavity in the female model than in the male model under these conditions.

#### 3.6.2. Delivery of MF@MOF with a Nozzle Angle of 30°

**Figure 9:**
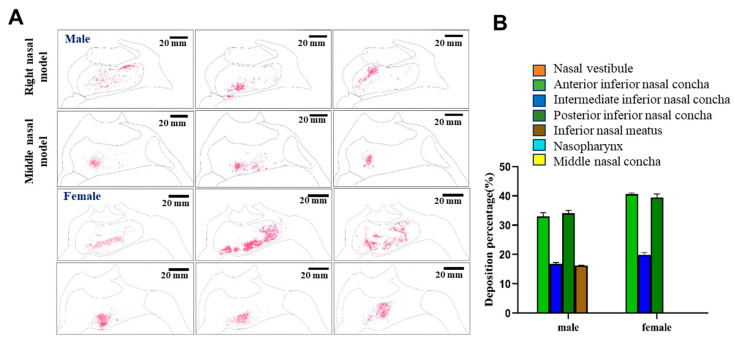
Nasal cavity deposition pattern of MF delivered as a nasal powder with an injection angle of 30°. The drug was delivered into 3D models mimicking the nasal cavities of male or female human subjects, and deposition of fluorescent MF was observed under UV irradiation (**A**). Semi-quantitative analysis of the fluorescence intensities shown in (**B**).

The distributions of powder in the models were also determined upon orienting the nozzle with a 30° cone angle (Figure 9). Under these conditions, no powder deposition was observed in the nasal vestibules of either model upon qualitative analyses. Instead, most of the drug was found to be delivered to the respiratory area and other more distal areas. But the specific distribution needed to be quantitatively calculated.

When the distributions of the average fluorescence intensities were investigated semi-quantitatively, the biggest difference between males and females was that 16.19% of the drug powder reached the inferior nasal meatus in the male model, while no drug was found to reach the inferior nasal meatus in the female model. Instead, with the nozzle at a 30° angle, all of the drug was delivered to the inferior turbinate region of the respiratory area in the female model, and most of the drug was delivered to the inferior turbinate and the inferior nasal canal of the male model. Overall, under these conditions, approximately 50.84% of the drug would be expected to reach the respiratory area, including the middle and posterior inferior turbinate, of male subjects, while approximately 59.38% of the drug would be expected to reach these areas in female subjects. In addition, taken together, these results suggested that a 45° nozzle angle would be optimal for delivery of the drug to the olfactory area in female subjects, while a 30° nozzle angle would be optimal for male subjects. It can be concluded that in both male and female models, a spray angle of 30° was more deposited in the inferior and middle nasal concha and less in the nasal vestibule, so 30° was more appropriate.

#### 3.6.3. Comparison of Delivery of MF@MOF to That of a Commercial MF Nasal Spray

**Figure 10:**
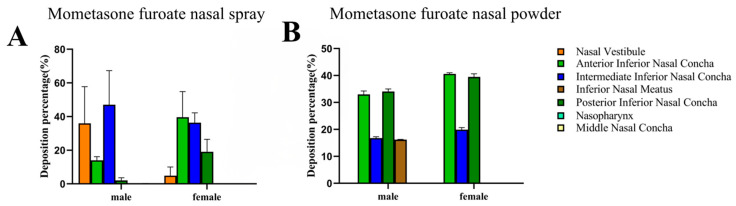
Comparison of the deposition of MF from MF@MOF to deposition from a commercial MF spray. The deposition of fluorescent MF upon application at an angle of 30° was measured using a 3D biomimetic model of the human nasal cavity. Nasal deposition distribution histogram of mometasone furoate nasal spray (**A**). The columnar diagram of deposition and distribution of mometasone furoate nasal powder spray (**B**).

We next compared the delivery performance of the powder described in this study with that of a commercially available MF spray using the biomimetic human nasal cavity models, both delivered using an angle of 30°. The drug would not remain in the nasal vestibular area when squeezed at 30°. A comparison of the distribution of drug deposition associated with the spray (Figure 10A) with that associated with the powder (Figure 10B) suggested that much of the drug delivered using the spray formulation was retained in the nasal vestibule, whereas very little of the powder-distributed drug was deposited in this area. As noted above, with the powder-based delivery system, approximately 50.84% of the drug was retained in the respiratory area of the male model, with 59.38% of the drug retained in the respiratory area of the female model. By comparing the percentage of deposition of spray and powder in the model, it was found that the spray would have retention in the nasal vestibule, while the powder would not. According to these results, we concluded that delivery of MF as a nasal powder led to a better drug distribution than did delivery of MF as a traditional nasal spray.

## 4. Discussion

Nasal drug delivery was first reported in the early 1980s as a potential alternative route for systemic drug administration, in contrast to conventional methods. Today, nasal administration is widely employed for the treatment of local upper respiratory disorders, such as nasal congestion, nasal infections, and allergic nasal diseases [40]. Chronic rhinosinusitis (CRS) is a heterogeneous condition characterized by local chronic inflammation of the mucous membranes of the nose and paranasal sinuses. Intranasal corticosteroid administration offers a rapid, straightforward, and cooperation-independent treatment approach, making it the preferred therapeutic option for patients. This method optimizes quality of life while simultaneously minimizing risks of disease progression and complications [41]. However, as a first-line drug for chronic rhinosinusitis treatment, mometasone furoate suffers from limited bioavailability due to its poor solubility. A CD-MOF, with its structural advantages, can provide abundant drug-loading sites for pharmaceutical molecules and has emerged as a powerful tool for enhancing the solubility of poorly water-soluble drugs. γ-CD possesses a larger cavity compared with β-cyclodextrin (β-CD), enabling it to encapsulate a broader range of guest molecules. Additionally, γ-CD exhibits superior water solubility. Therefore, γ-CD was chosen as the organic ligand for CD-MOF synthesis [42].

We successfully fabricated CD-MOF carriers with particle sizes ranging from 12 to 152 μm, which met the particle size requirements for nasal powder spray. During the drug loading process, three types of non-aqueous solvents were systematically evaluated, with anhydrous ethanol ultimately being identified as the optimal solvent for drug dissolution. The successful encapsulation of drug molecules within the CD-MOF cavities was thoroughly characterized and confirmed using three complementary analytical techniques: powder X-ray diffraction (PXRD) patterns, thermogravimetric analysis (TGA) thermograms, and synchrotron radiation Fourier transform infrared (SR-FTIR) spectra. By loading MF into the cavity of the CD-MOF, the solubility of MF was significantly improved according to the vitro solubility studies. In vitro release studies demonstrated that the solubility of MF@MOF in artificial nasal solution increased to 162.5 mg/mL, representing an approximately 800-fold enhancement compared with free MF. Moreover, the release profile exhibited pH-independent behavior, achieving an 85% release rate within 5 min in water or other media, which was significantly superior to that of the physical mixture (only 12%). Due to the facile dissociation of CD-MOFs in aqueous media (particularly K⁺-based CD-MOFs), cyclodextrin–drug inclusion complexes or free drug molecules were released, while the dissociated cyclodextrins continued to exert solubilizing effects. The hydrophobic cavity of cyclodextrins could encapsulate the hydrophobic moieties of poorly soluble drugs through van der Waals forces, hydrophobic interactions, etc., forming soluble inclusion complexes (analogous to conventional cyclodextrin complexation techniques), thereby enhancing the hydrophilicity of the drug molecules. Therefore, it was considered that MF, upon being encapsulated within the hydrophobic cavity of cyclodextrin, formed a soluble inclusion complex, leading to a significant improvement in the solubility of MF. Subsequently, in the vitro release study, a release medium volume of 5 mL was selected based on two considerations: First, as the prepared nasal powder formulation primarily targeted local action with direct drug delivery to the nasal mucosa rather than systemic absorption, the 5 mL volume better approximated the actual physiological capacity of the nasal cavity [43]. Second, the release profile indicated that this formulation represented a rapid-release system (complete release within the first 5 min), rendering sink conditions less critical for this evaluation.

The deposition pattern of nasal formulations in the nasal cavity determines both the effective absorption amount and the site of drug action. For innovative nasal powder formulations in particular, targeted delivery to specific nasal regions must be optimized to ensure therapeutic efficacy. Current pharmacopoeial standards for nasal preparations primarily focus on the total delivered dose, specifying metrics such as dose per spray and delivered dose per actuation, yet fail to provide evaluation methods for regional drug deposition. A three-dimensional biomimetic model of the human nasal cavity, developed by our team using three-dimensional reconstruction and 3D printing technologies based on computed tomography (CT) data from 32 Chinese adults, holds significant value for evaluating the deposition and distribution patterns of nasal formulations. Using this model, we investigated drug distribution patterns in both male and female nasal cavities at 45° and 30° administration angles. Comprehensive analysis revealed that 30° administration resulted in preferential drug deposition in the middle-posterior segments of the nasal cavity, which represented the optimal distribution pattern for our purposes. Subsequent comparison with commercially available mometasone furoate nasal spray demonstrated that our self-developed nasal powder spray achieved significantly better distribution in the middle-posterior nasal regions, whereas the commercial product primarily accumulated in the anterior nasal cavity, suggesting better pathological targeting capability.

## 5. Conclusions

In this study, MF was successfully loaded into the cavities of CD-MOFs via an incubation method, yielding MF@MOF with a drug loading content of approximately 4%. The influence of different solvents on drug loading capacity was further investigated. Subsequent in vitro release experiments conducted in four distinct release media provided additional evidence for the successful encapsulation of MF within CD-MOF cavities. In vitro deposition experiments indicated that the effect was best when the nozzle was sprayed at 30°, and the powder deposition in the nasal vestibule was the least. More importantly, the distribution of nasal powder sprays reached more into the respiratory and olfactory regions, while most of the sprays remained at the nasal vestibule. Only when nasal drugs were delivered to the target area of the nasal cavity could their efficacy be exerted. This study pioneered the integration of CD-MOF carriers with a 3D biomimetic model of the human nasal cavity, offering a dual innovative approach for a nasal delivery system design: on one hand, CD-MOFs address the delivery challenges of poorly soluble drugs; on the other hand, 3D printing technology enables the simulation of drug deposition and distribution behavior in nasal drug delivery. Future research should further investigate the stability of MF@MOF, as well as its anti-inflammatory efficacy and in vivo pharmacokinetics in CRS animal models, thereby laying the foundation for the clinical translation of nasal powder formulations.

## Figures and Tables

**Table 1:** Single-factor study of the effects of incubation parameters on drug loading efficiency.

Solvent	MF/CD-MOF Ratio, % (*w*/*w*)	Loading Efficiency, % (*w*/*w*)
Acetone	4.55	4.22
	5.88	5.36
	8.70	8.23
	4.55	4.46
Ethyl acetate	5.88	5.57
	8.70	8.63
	4.55	4.25
Anhydrous ethanol	5.88	5.63
	8.70	8.21

## Data Availability

Data are included within the article.

## Abbreviations

The following abbreviations are used in this manuscript:

MF	Mometasone furoate
CD-MOF	Cyclodextrin metal-organic framework
CRS	Chronic rhinosinusitis
γ-CD	γ-Cyclodextrin
3DP	Three-dimensional printing
SEM	Scanning electron microscopy
SR-FTIR	Synchrotron radiation Fourier transform infrared spectroscopy
PXRD	Powder X-ray diffraction
TGA	Thermogravimetric analysis
